# Sequence Polymorphism in Xenobiotic Metabolising Genes in Iraqi Colorectal Cancer Patients

**DOI:** 10.31557/APJCP.2021.22.4.1203

**Published:** 2021-04

**Authors:** Salih Q Ibrahem, Zana T Al-Dalawi, Assma S Bahaaldin

**Affiliations:** 1 *Department of Biochemistry, College of Medicine, Kirkuk University, Iraq. *; 2 *Department of Pathology, Azadi Teaching Hospital, Kirkuk, Iraq. *; 3 *Clinical Oncologist, Kirkuk Oncology Centre, Kirkuk, Iraq. *

**Keywords:** Colorectal cancer, cytochrome, CYP1A1, CYP1B1, polymorphisms

## Abstract

**Objectives:**

Colorectal cancer (CRC) is the third most prevalent malignant neoplasm. Genetic variations in the xenobiotic metabolising cytochrome enzymes. Family 1 Subfamily A Member 1 (*CYP1A1*) and Family 1 Subfamily B Member 1 (*CYP1B1*) might play a role in cancer pathogenesis and prognosis. The aim of this work is to determine the frequency of Single Nucleotide Polymorphisms (SNPs) in *CYP1A1* (rs1048943, Ile462VaI and rs4646903/MSP1) and CYP1B1 (rs1056836, Leu432Val) genes in patients with CRC cancer. It was also an attempt to identify the association between SNPs and CRC and its stage and grade at diagnosis.

**Methods:**

This case-control study was conducted in Kirkuk/Iraq, 200 patients with CRC and 200 cancer free control subjects were enrolled. Genomic DNA was extracted from venous blood samples and screened for SNPs using Restriction Fragment Length Polymorphism (RFLP) and confirmed by the direct DNA sequencing.

**Results:**

The reference genotype of *CYP1A1* gene rs1048943 is AA. Both the AG and GG variants were significantly more frequent in the cancer group and associated with increased risks of CRC and its later stages (stages III and IV) and poor differentiation (p<0.01). The reference genotype of CYP1A1 rs4646903 is TT. The variant genotypes, TC and CC, had no significant association with increased odds of cancer (P>0.05) or with tumour stage or its grade (p>0.05). The GG genotype of CYP1B1 rs1056836 was the reference genotype. The CG and CC variants were not associated with increased risks of CRC (P>0.05) or its stage or grade except the CG genotype which was associated with poor differentiation (OR= 3.4, 95 % CI= 1.8 -6.5, P<0.001).

**Conclusion:**

CYP1A1 gene rs1048943 SNPs can represent a potential future marker for CRC risk prediction and prognosis. Further evaluation in large scale studies will provide greater understanding of the effects of other genes SNPs on CRC risk and prognosis.

## Introduction

Colorectal Cancer (CRC) is the 3^rd^ most frequent type of malignancy and the 4^th^ leading cause of cancer morbidity worldwide, with almost ten difference in the incidence and mortality between the Western and Eastern parts of the world (Arnold et al., 2017; Siegel et al., 2020). The global burden of colorectal cancer (CRC) is expected to increase by 60% to more than 2.2 million new cases and 1.1 million deaths by 2030 (Arnold et al., 2017).

CRC results from interaction of different factors that disrupt the fine and delicate genetic make-up of cells (Rudolph et al., 2016). These factors are hereditary, familial and environmental, the last two factors are estimated to be responsible for 70% of CRC cancer cases in the USA (Jemal et al., 2010). One of the environmental factors is the exposure to xenobiotics (Croom, 2012). Xenobiotics have been defined as foreign chemical substances to that living organism that are not part of its natural metabolic process and would be harmful if not properly metabolised (Croom, 2012). Xenobiotics include pollutants, food additives, oil mixtures, pesticides, synthetic polymers, carcinogens and Polycyclic Aromatic Hydrocarbons (PAHs) (Qadir et al., 2017). 

Polycyclic Aromatic Hydrocarbons xenobiotics (PAHs) are a spectrum of compounds with a structure of fused benzenoid rings (alternant PAHs), in addition to unsaturated four-, five-, and six-membered rings (non-alternant PAHs). PAHs are produced by petroleum and gas industries and the use of fossil fuel and smoking (Boström et al., 2002). Areas and cities with large petroleum industries are well known to have higher level of PAHs in their air, sediment and water (Tiwari et al., 2011; Asagbra et al., 2015; Keshavarzi et al., 2015). PAHs have been recognised by the world health (WHO) organisation as ‘probably carcinogenic to humans’ (Group 2A) that are activated to carcinogens via cytochrome P450 enzymes through phase I reactions (Shimada and Fujii-Kuriyama, 2004; Jameson, 2009). 

Cytochrome P450 enzymes are of four families (CYP1-CYP4). Family 1 Subfamily A Member 1, previously known as aryl hydrocarbon hydroxylase, (CYP1A1, 15q24.1) and Cytochrome P450 Family 1 Subfamily B Member 1 *(*CYP1B1, 2p22.2) monooxygenases were shown to be particularly involved in PAHs metabolism (Shimada and Fujii-Kuriyama, 2004). There is little hepatic expression of *CYP1A1* and CYP1B1 that occurs mostly in non-hepatic tissues such as the gastrointestinal tract and breast (Masson et al., 2005; Androutsopoulos et al., 2013). 

Both enzymes activate PAHs to carcinogens through phases I reaction to epoxides which are converted by epioxide hydrolase enzyme to more reactive diol-epoxides (Shimada and Fujii-Kuriyama, 2004; Shimada, 2006). The later compounds can cause extensive DNA damage in the form of DNA adducts with subsequent gene mutations that are essential to carcinogenesis (Ewa and Danuta, 2017). PAHs can, interestingly, induce these enzymes gene expression which will generate a vicious circus of PAHs activation (Iwanari et al., 2002).

It was interesting to find that there is a significant difference in the risk of CRC between different populations who have the same SNP (Wang et al., 2011; He et al., 2014). Better understanding of SNP in these enzymes will improve our understanding of the mechanisms of the disease and facilitate the design of effective population specific risk-assessment models. 

The importance of CYP1A1 and CYP1B1 cytochrome enzymes genotyping in Kirkuk governorate stems from its massive petroleum industry and high level PAHs that are metabolised by the earlier cytochrome enzymes. CYP1A1 and CYP1B1 genetic variations might change enzymes functional characteristics in PAHs metabolism and ultimately influence the risk of CRC, which is increasing in Kirkuk governorate (Al Dahhan and Al Lami, 2018).

In this work, the aim is to identify whether polymorphisms in *CYP1A1* and* CYP1B1* genes increase the susceptibility of Iraqis in Kirkuk governorate to CRC. The first polymorphism is an A/G transition (rs1048943) in exon 7 of the *CYP1A1* gene which changes Ile462Val and the second is the non-coding SNP rs4646903 of CYP1A1. The third is G/C transversion (rs1056836) of *CYP1B1 *gene which changes Leu432Val. 

## Materials and Methods

*Subjects*

This case control study was conducted at Kirkuk Oncology Centre (KOC), Kirkuk, Iraq. It encompasses two hundred patients with histopathology confirmed CRC of the adenocarcinoma type who visited the centre for treatment or follow up during the period from 1^st ^June 2019 till 1^st^ October 2020. Two hundred cancer free matched age and sex subjects were chosen as controls. The participant age range was 30-73 years. The study ethical approval was obtained from Kirkuk Medical College Ethics Committee. Verbal or written consents were taken from each participant after a full explanation of the research aims and risks. Cases with known family history of cancer were excluded from the study. 

A questionnaire has been constructed and all the participant clinico-pathological characteristics were taken including age, sex, marital status, educational level, occupation, history of chronic disease and family history of cancer. In addition to the histopathological type, stage and grade of the tumour at diagnosis according to the TNM staging system (Amin et al., 2017).

*Blood samples, DNA extraction and Polymerase Chain Reaction*

Five millilitre (ml) of peripheral venous blood were obtained from each participant and placed in EDTA tube and DNA was purified using the QIAamp DNA Blood Mini Kit^®^ (Cat. No. 51104, Qiagen, GmbH) following the supplier recommendations. Previously used primers were used for the Polymerase Chain Reactions (PCRs) for genotyping of SNPs of CYP1A1 (rs1048943 and rs4646903) and *CYP1B1* (rs1056836) cytochrome genes. The source of the primers and their sequences are shown in Table 1. 

The PCRs had a final volume of 25 microliter (µl) consisting of 20nanogram (ng) of DNA, 10µl of PCR master mix (Maxime PCR PreMix kit, Cat. No. 25025, iNtRON^®^ Bioyechnology), 10picomol (1µl) of each primer and the reaction were brought up to the final volume with molecular grade water. MultiGeneOptiMax Gradient Thermal Cycler^®^ (Lab net, USA) was used according to the following: initial denaturation phase at 94°C for 5 minutes followed by 40 rounds of 94°C/62°C/72°C each for 45 seconds, the final phase was the extension which lasted for 7 minutes at 72°C. 

*SNP identification and confirmation*

Restriction Fragment Length Polymorphism (RFLP) method was used for SNPs genotyping using restriction enzyme specific for digestion of each PCR product in a total volume of 10ul as clarified in Table 2. The digested PCR products were loaded on 2% agarose gel and separated then visualised under UV light (Vilber lourmat®, France). The digestion condition of each PCR product, the expected sizes of the digestion products and the amino acid changes for each genotype are shown in Table 2. Eighteen samples were sent for direct sequencing, the genotyping gold standard, to Macrogen^®^ Company (Seoul, Korea). Sequencing results were viewed and analysed using Chromas 2.6.6 software (Technelysium Pty Ltd, Australia). 

*Statistical analysis*

The analysis of the data was done by the GraphPad Prism 8^®^ software (San Diego, CA, USA). The Chi Square test (with fisher’s exact test to compute P value) was used to calculate the OR (odds ratio) and 95% CIs (confidence intervals) for the determination of the association between polymorphisms and cancer risk. SNP frequency differences between the cancer and control subjects were estimated by the Fisher’s exact test. Quantitative (numerical) parameters were analysed by unpaired T Test (Student’s T-Test). P value <0.05 was recognised as statistically significant.

## Results

*Clinico-demographic characteristic*

The patients had an age range of 21-91 years (mean 56 years) while for the control it was 27-79 years (mean 53 years). No statistically significant difference was found between the patients and the control groups regarding the mean age, sex, educational level (p<0.05) as clarified in Table 3. Anatomical location, stage and grade are also shown. The study subjects followed Hardy-Weinberg equilibrium (HWE) for the tested genotypes.

*Associations of SNP genotype variants with CRC risk*

**Figure 1:**
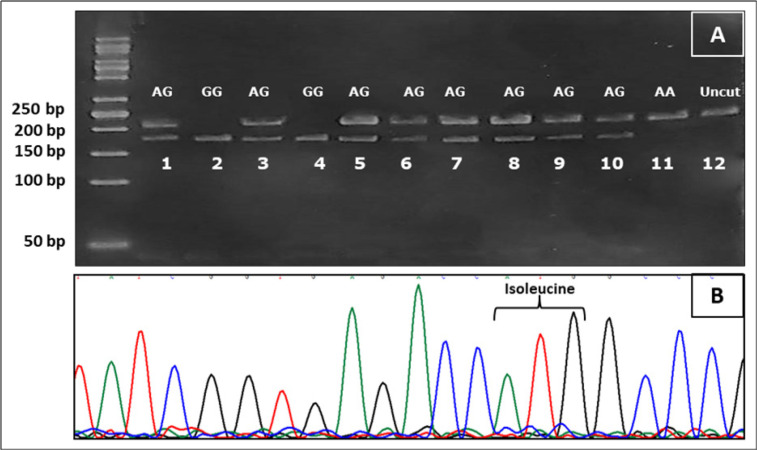
Screening for *CYP1A1* rs1048943 Genotypes Using RFLP Technique and Confirmation by Direct Sequencing. (A), RFLP for *CYP1A1* rs1048943 is determined by variations in the size of the digested fragments. Lanes: 1, 3, 5, 6, 7, 8, 9 and 10 show two bands of 212 and 190 base pair (bp) representing AG genotype, while lanes 2 and 4 show single band of 190bp of GG genotype. Finally lane 11 shows a single 212bp band which corresponds to homozygous AA genotype; (B), Direct sequencing showing homozygous AA genotype (the same sample as in lane 11) which codes for Isoleucine (ATG). Lane 12 is a control lane (Uncut) with no restriction enzyme added and a single band of 212bp

Examples of SNP RFLP and direct sequencing are shown in Figures: 1 (CYP1A1 rs1048943), 2 (CYP1A1 rs4646903) and 3 (CYP1B1 rs1056836). CYP1A1 rs1048943 SNP genotyping shows that the AA genotype is the reference genotype (common genotype) in both: control (69%) and CRC (56%) groups. The AG variant was more common in the cancer group (33%) than the control one (25%) and was significantly correlated with increased risks (p=0.03) of CRC. The GG variant of *CYP1A1 *gene rs1048943 SNP increased the cancer risk by more than two folds. Numerical details in depicted Table 4. 

The genotype TT od CYP1A1 rs4646903 was the most common genotype in both the control and patient subjects (50% and 47%) respectively, with no significant differences. The TC genotype was the second most common and identified in almost one third of both groups (control, 33% and patients, 30%) with no significant correlation with increased risks of CRC (p>0.05) as shown in Table 4. The CC genotype showed no statistically significant correlations with CRC (p>0.05), Table 4. 

The frequency of genotypes of *CYP1B1* gene (rs1056836) among the 200 patients was CC (64.0%), CG (33%) and GG (3%), while in the control group it was CC (71%) CG (28%) and GG (1%), as can be seen in Table 4. All these variants do not impose any significant increased risk of CRC to their carriers.

*Variant genotypes association with CRC stage*

In this work, stages I and II (with all their sub-stages) were considered early stages while stages III and IV( with all their sub-stages ) were recognised as late stages. Both genotypes of *CYP1A1* rs1048943 (AG and GG) were significantly associated (P<0.01) with late stages of CRC (III and IV) in comparison to the most common (reference) genotype: AA. None of the genotypes (TC and CC) of the *CY1A1 *(rs4646903) showed any significant association (p>0.05) with the stages of the disease. *CYP1B1* rs1056836 genotypes (CG and CC) revealed no significant association with the stages of CRC relative to the common genotype (GG). Details of the statistical analysis of all the SNPs and their genotypes are shown in Table 5. 

*Variant genotypes association with CRC grade*

Grades I and II were considered one category, while grade III was considered another (poorly differentiated). Briefly, GG and AG variant genotypes of CYP1A1 rs1048943 had strong associations with poor differentiation. All the genotype of the other SNP of CYP1A1 rs4646903 revealed no associations with the grade of the tumour. 

The CC genotype of CYP1B1rs1056836 did not reveal any statistically significant correlations with the grade of the tumour, while the CG variant had a strong correlation with the poorly differentiated tumours. Table 6 contains details of the percentages of the genotypes grade and the degree of association.

**Figure 2:**
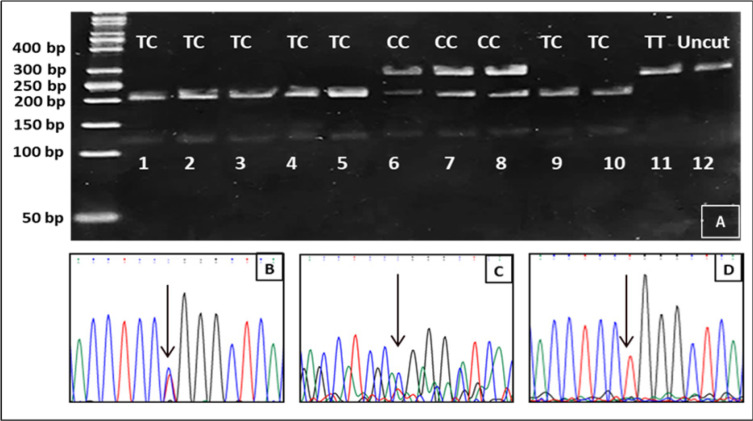
The RFLP and direct sequencing of SNPs in *CYP1A1* rs4646903. (A) Lanes:1,2,3,4,5,9,and 10 showing two bands of 209 and 133 for TC genotype and confirmed for sample in lane 3 in the direct sequencing seen (black arrow) in picture (B). Three bands are present in lanes: 6,7 and 8 of 342,209 and 133bp for the CC genotype which is seen in the direct sequencing chromatogram(black arrow) in (C). Lane 11 is a homozygous variant (TT) of the SNP that is verified by direct sequencing in (D) which shows a single signal of thymine (black arrow). The last lane (12) is a control sample with no added restriction enzyme and has a single band of 342bp

**Table 1:** The Genes, SNP Reference Sequence (rs) and Their Synonyms, Exon where the SNPs are Located and the Sequences of the Primers and Their Sources

The gene	SNP rs ID	Exon	Forward primers	Reverse primers	Reference of the source of the primers
*CYP1A1*	rs1048943 (VAR_001243, rs386513458, rs52810784, rs3188998, rs17861092 )	7	5′CCACTCACTTGACACTTCTGAGCCC 3′	5′AAAGACCTCCCAGCGGGCCA 3′	(Ding et al., 2017)
*CYP1A1*	rs4646903(rs17861083, rs5030838, rs116877783)	Un-translated	5’-CAGTGAAGAGGTGTAGCCGC-3’	5’-TAGGAGTCTTGTCTCATGCC3’	(Vijayalakshmi et al., 2005)
*CYP1B1*	rs1056836	3	5`CACCACTGCCAACACCTCTGTC3	5'-AGTTCTCCGGGTTAGGCCACTTAA-3	(Gehan A. El-Shennawy and Elbehery, 2010)

**Table 2:** Digestion Enzymes, Their Supplier, Digestion Conditions, the Size of the Digested Fragments in Base Pair (bp) and the Amino Acid Changes

The SNP	Restriction enzyme	Digestion conditions	Products size	Amino acid
"*CYP1A1* rs1048943 ILe 462Val"	EcoT14I (StyI)	5µl (PCR product) 1µl (enzyme) 4µl (buffer) 1hour at 37°C	212bp (AA)	Isoluecine
"*CYP1A1* rs4646903 "	(TaKaRal®/ Japan)	5µl(PCR product) 1µl (enzyme) 4µl (buffer) 1hour at 37°C	190,212bp (AG)	Isoleucine/Valine
"*CYP1B1* rs1056836 Leu 432Val "	MspI (HapII , HpaII) (TaKaRal®/ Japan)	5µl (PCR product) 1µl (enzyme) 4µl (buffer) 1hour at 37°C	190 bp (GG)	Valine

**Table 3:** The Clinico-Pathological Characteristics of the Study Subjects, CRC Patients (n=200) and Control Subjects (n=200)

Characteristic	CRC Patients	Controls	P value
Total no.	200	200	
Mean age ± SD, years	56±35	53±26	˃0.05
Sex			
Male	116 (58%)	112 (56%)	˃0.05
Female	84 (42%)	88 (44%)	˃0.05
Education level			
Up to high school	134 (67%)	126 (63%)	˃0.05
Beyond high school	66 (33%)	74 (37%)	˃0.05
Location of tumour			
Rectum	90 (45%)		
Left Colon	60 (33%)		
Right Colon	44 (22%)		
TNM stage			
I	25 (12.5%)		
II	79 (39.5%)		
III	74 (37%)		
IV	22 (11%)		
Differentiation			
Well-differentiated	30 (15%)		
Moderately differentiated	104 (52%)		
Poorly differentiated	66 (33%)		

**Figure 3:**
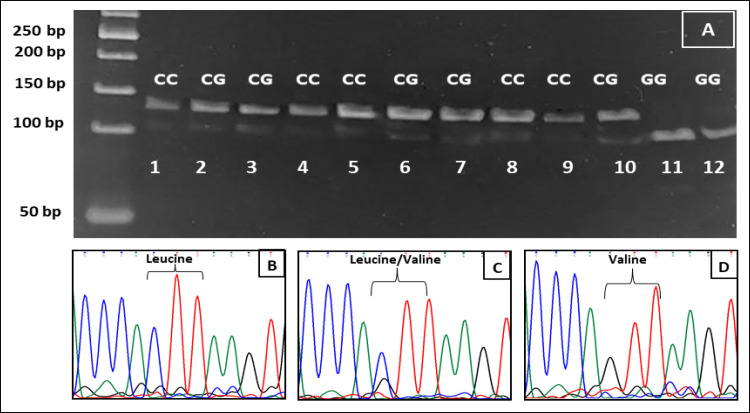
RFLP and Sequencing Result of *CYP1B1* rs1056836. Lanes: 1,4,5, 8 and 9 have two bands of 113 and 135bp for the CC genotype, direct sequencing of this genotype shown in (B) which codes for Leucine. Lanes: 2,3,6,7 and 10 depict two bands of 108 and 135bp of the CG genotype which was confirmed by direct sequencing as shown in (C) which codes for Leucine/Valine. Lanes 11 and 12 of the gel have a single band of 108bp that reflects GG genotype as confirmed by direct sequencing in (D) that codes for Valine

**Table 4:** Genotypes frequencies of CYP1A1 rs1048943, CYP1A1 rs4646903 and CYP1B1 rs1056836 in both the control (n=200) and patient subjects (n =200) and their association

Gene SNPrs	Genotype	Control no, %	Patients no, %	OR (95%CI)	P-value
*CYP1A1 *rs1048943	AA	138 (69 %)	112 (56%)	1 (Reference)	
	AG	50 (25 %)	66 (33%)	1.7 (1.0 -2.5 )	0.03
	GG	12 (6%)	22 (11 %)	2.3 (1.1-4.8)	0.03
*CYP1A1* rs4646903	TT	100 (50%)	94 (47%)	1 (Reference)	
	TC	66 (33 %)	60 (30%)	1.2 (0.7 -1.8 )	0.6
	CC	34 (17%)	46 (23%)	1.4 (0.9-2.4)	0.2
*CYP1B1* rs1056836	GG	142 (71%)	128 (64%)	1 (Reference)	
	CG	56 (28%)	66 (33%)	0.7 (0.5 - 1.2)	0.2
	CC	2( 1.0%)	6 (3%)	0.3 (0.1 -1.3)	0.2

no, number of subjects; OR, Odds Ratio; CI, confidence interval

**Table 5:** Genotype Frequencies of *CYP1A1*rs1048943, *CYP1A1*rs4646903 and *CYP1B1* rs1056836 in Patient Subjects (n =200) and Their Associations with the Tumours Stage. The number and the percentage are within the same genotype

Gene		Stages		OR (95%CI)	P-value
	Genotype /Total number	I& II no (%)	III& IV no(%)		
*CYP1A1* rs1048943	AA (112)	73 (65.2%)	39 (34.8% )	1(Reference)	
	AG (66)	23 (34.8% )	43 (65.2%)	3.5 (1.8- 6.4)	<0.0001
	GG (22)	8 (40.9 % )	14 (59.1%)	3.3 (1.3 -8.4)	0.01
*CYP1A1* rs4646903	TT (94)	45 (47.9%)	49 (52.1%)	1(Reference)	
	TC (60)	34 (56.7%)	26 (43.3 %)	1.7 (0.9 -3.2 )	0.1
	CC (46)	25 (54.3%)	21 (45.7%)	0.8 (0.4-1.6)	0.6
*CYP1B1* rs1056836	GG (128)	69 (53.9%)	59 (46.1%)	1(Reference)	
	CG (66)	32 (51.5%)	34 (48.5%)	1.2 (0.7 -2.3 )	0.5
	CC (6)	3 (50.0%)	3 (50.0%)	1.2 (0.3-5.2 )	>0.9

**Table 6:** Genotype Frequencies of *CYP1A1*rs1048943, *CYP1A1*rs4646903 and *CYP1B1* rs1056836 in Patient Subjects (n =200) and Their Association with Tumours Grade. The number and the percentage are within the same genotype

Gene		Grade/differentiation		OR (95%CI)	P-value
		Well & moderate differentiation no (%)	Poor differentiation, no (%)		
*CYP1A1* rs1048943	AA (112)	95 (74.1%)	17 (25.9% )	1(Reference)	
	AG (66)	30 (34.8% )	36 (65.2%)	6.7 (3.2-13.5 )	<0.0001
	GG (22)	9 (22.7 % )	13 (77.3%)	9.0 (2.9 -21.4 )	<0.0001
*CYP1A1* rs4646903	TT (94)	61 (64.9%)	33 (34.1 %)	1 (Reference)	
	TC (60)	43 (71.6%)	17 (28.4 %)	0.7 (0.4-1.5)	0.48
	CC (46)	30 (65.2%)	16 (34.8%)	0.98 (0.5-2.1)	>0.99
*CYP1B1* rs1056836	GG (128)	99 (77.3%)	29 (22.7%)	1 (Reference)	
	CG (66)	33 (50.0%)	33 (50.0%)	3.4 (1.8 -6.5)	<0.001
	CC (6)	2 (33.3)	4 (67.7)	6.8 (1.5 -36.6 )	0.2

## Discussion

In this work, an attempt was made to identify genetic variants that make people more susceptible to CRC, especially those in the cytochrome genes that play a role in phase I PAH metabolism namely; *CYP1A1* (rs1048943 and rs4646903) *CYP1B1* (rs1056836) (Ghisari et al., 2014).

*CYP1A1* (rs1048943) is a hot spot for genetic polymorphism, the transition of the common AA genotype, codes Isoleucine, to AG and GG variants results in coding for Ioleucine/Valine and Valine/Valine respectively. The latter two variants are associated with increased risks of CRC in this work, this finding is justifiable. These changes are associated with increased expressions and activities of the coded enzyme and consequently DNA is damaged due to the increased free radical generation from the activation of PAH (Shimada and Fujii-Kuriyama, 2004; Masson et al., 2005). 

Two meta-analysis reviews that looked at the association of CYP1A1( rs1048943) with CRC in 26 case control studies found conflicting results (Chen et al., 2005; Zhu et al., 2016). One Korean study revealed no association between genotypes and increased risks (Cho et al., 2017), while there was a positive association between the variants and increased odd of CRC cancer in some Asian and European people (Kobayashi et al., 2009; Zheng et al., 2009; Li et al., 2015).

The controversy in the relation can be attributed to the fact that occurrence of cancer is not a simple cause and effect relation. There is a large number of players in the field such as the genome as a whole and its interaction with other in vivo and environmental factors. 

It was interesting to find, in the current work, that the above variant genotypes are associated with poor prognosis since higher tumour stages and poor differentiations were more common in the patients harbouring the variants when compared to common genotype. The mechanism by which these variants influence the stage and the grade is yet to be identified. However, this relation seems to be racially and cancer type is modified. For example, an Iranian breast cancer study showed some associations of the variants with cancer grade but not with breast stage (Bab Rahmati et al., 2016). On the contrary, a Brazilan research found that Ile462Val is not associated with stage or grade of CRC (Serafim et al., 2008). Another study found that these variants are associated with better drug response in breast cancer (Dong et al., 2012).

*CYP1A1* rs4646903 SNP (MspI) polymorphism, in this work, (TC, CC) imposed no increased risk when compared to the common genotype (TT). A meta-analysis of 26 studies (from different geographic and ethnic settings) did not identify any association between the genotype variation and the risk of CRC (He et al., 2014). This analysis contradicts a Chinese study which observed a reduced CRC in those harbouring these genotype (Kamiza et al., 2018). However, Saudi and Japanese studies showed increased risk (Nisa et al., 2010; Saeed et al., 2013). The effect of the genotypes on cancer risk, despite being non coding, can be due its link with another polymorphism (linkage disequilibrium) or these variants may influence other gene activities by altering their messenger *RNA *expression level, location and stability (Schmitt and Chang, 2016). *CYP1A1* rs4646903 SNP (MspI) polymorphism has been associated, in vitro, with greater catalytic activity and consequent carcinogen activation (Landi et al., 1994).

SNP rs1056836 G/C transversion results in Leucine being replaced by Valine at codon 432 which is located at heme-binding domain. The Valine amino acid increases the activity of the CY1B1 enzyme which exhibit greater catalytic 4-hydoxylation activity than the wild type enzyme (Trubicka et al., 2010). A British study did not show any correlation between the SNP and CRC risk (Bethke et al., 2007), similar observations were in Caucasians (Xie et al., 2012) while a polish study showed a correlation with CRC (Trubicka et al., 2010). While in Czech population the variants showed a protective effect (Hlavata et al., 2010)

These studies do not have an exactly similar study design, age of population, confounders and inclusion and exclusion criteria. In this work, no statically significant association was identified between CYP1B1 genotypes with neither odd of cancer risk nor with its stage or grade, except for the CC genotype, which is associated with poor differentiation.

To sum the discussion up, each population with distinct ethnic and geographic characteristics would have a different correlation of their genotypes with CRC cancer risk and severity. 

In conclusion, genotypes of the CYP1A1 rs1048943 has a strong correlation with the occurrence of CRC cancer and its severity, therefore they might have a role in the design of future tool for the prediction of CRC risk and facilitate diagnosis. These tools, hopefully, will encourage the health care system to take a swift medical action for those patients with CRC who carry the variants that are associated with late stage and poor tumour differentiation. 

## Author Contribution Statement

Salih Q Ibrahem: Collection of blood samples and doing molecular test, performing the statistical analysis, writing up the paper. Zana T AL-Dalawi: Providing the hsitopathological data of the cases, helping with the writing up and the laboratory results. Assma S Bahaaldin: Selection of the cases and providing the clinco- demographic data of the cases 
